# Temperature can shape a cline in polyandry, but only genetic variation can sustain it over time

**DOI:** 10.1093/beheco/arv172

**Published:** 2015-10-25

**Authors:** Michelle L. Taylor, Tom A.R. Price, Alison Skeats, Nina Wedell

**Affiliations:** ^a^Centre for Ecology and Conservation, College of Life and Environmental Sciences, Biosciences, University of Exeter, Penryn Campus, Penryn, Cornwall TR10 9FE, UK and; ^b^Institute for Integrative Biology, Faculty of Health and Life Sciences, University of Liverpool, Crown Street, Liverpool L69 7ZB, UK

**Keywords:** *Drosophila*, *pseudoobscura*, environmental drivers, female behavior, genetic variation, sexual selection.

## Abstract

Multiple mating by females (polyandry) is a widespread behavior occurring in diverse taxa, species, and populations. Polyandry can also vary widely within species, and individual populations, so that both monandrous and polyandrous females occur together. Genetic differences can explain some of this intraspecific variation in polyandry, but environmental factors are also likely to play a role. One environmental factor that influences many fundamental biological processes is temperature. Higher temperatures have been shown to directly increase remating in laboratory studies of insects. In the longer term, high temperature could also help to drive the evolution of larger-scale patterns of behavior by changing the context-dependent balance of costs and benefits of polyandry across environments. We examined the relative influence of rearing and mating temperatures on female remating in populations of *Drosophila pseudoobscura* that show a latitudinal cline in polyandry in nature, using a range of ecologically relevant temperatures. We found that females of all genotypes remated more at cooler temperatures, which fits with the observation of higher average frequencies of polyandry at higher latitudes in this species. However, the impact of temperature was outweighed by the strong genetic control of remating in females in this species. It is likely that genetic factors provide the primary explanation for the latitudinal cline in polyandry in this species.

## Lay Summary

Cooler temperatures can cause female insects to mate more often, but genetic background still has strong control over long-term behavior. How often females mate has major impacts on conflict, cooperation, and how disease spreads within populations. We found that females mate more often when it is colder, which explains why females in northern populations mate more often than in southern ones. However, genetic background strongly determines the long-term patterns of female behavior within and across populations.

## INTRODUCTION

Polyandry, or mating with multiple males by females, is a widespread behavior, occurring in a diverse array of taxa from a variety of ecological and environmental habitats (Andersson 1994; Birkhead and Møller 1998; Taylor et al. 2014). However, within polyandrous species, there remains considerable variation in the proportion of females routinely mating with multiple males, from as few as 10% to over 90% (Wedell 2005; Taylor et al. 2014). Females who mate multiply may be advantaged over singly mating females in numerous aspects of their reproductive biology (Arnqvist and Nilsson 2000; Jennions and Petrie 2000; Simmons 2001, 2003, 2005). However, the costs and benefits of polyandry are likely to be context dependent, with environmental influences contributing to the background selection on polyandry (Candolin and Heuschele 2008; Gowaty 2012, 2013). One environmental influence on female remating that has recently been of interest is the effect of ambient temperature (Saeki et al. 2005; Kindle et al. 2006; Katsuki and Miyatake 2009; Kellermann et al. 2009; Olsson et al. 2011; Grazer and Martin 2012; Best et al. 2012).

Temperature may influence female remating directly by increasing the speed and energy of poikilothermic species. Temperature may also change the balance of costs and benefits over successive mating episodes. For example, thermally regulated metabolic functions affect the energy available for courtship and mating behavior in both males and females (Hoffmann et al. 2003). High temperatures can also impose time constraints on courtship and mating through risk of desiccation (Kellermann et al. 2009; Grazer and Martin 2012), or increase the rate at which important proteins denature, such as the sex peptides secreted and transferred at mating by many male insects to reduce female remating (Best et al. 2012). Temperature may also indirectly affect female remating by influencing the operational sex ratio, as occurs in sand lizards where more individuals are more active at higher temperatures thereby increasing opportunities for mate encounter (Olsson et al. 2011). Many studies have so far shown a positive relationship between ambient temperature and remating, with females remating more at higher temperatures. For example, remating is more common at higher temperatures (33 vs. 17 °C) in the adzuki bean beetle, *Callosobruchus chinensis* (Katsuki and Miyatake 2009) and the Japanese beetle *Popillia japonica* (Saeki et al. 2005). *Drosophila melanogaster* females also show higher tendency to remate at higher temperatures than cooler temperatures (25 vs. 18 °C) (Best et al. 2012). Females remate more at higher temperature in crickets, with this single factor accounting for up to 50% of the variation in female remating behavior (Kindle et al. 2006). Temperature can also indirectly influence female remating by generating phenotypic variation in traits such as body size (Forster et al. 2011; Forster and Hirst 2012), which in turn affect important aspects of fitness such as survival, fecundity, and mating success (Kingsolver 2009). For example, females developing at cooler temperatures and maturing at larger sizes, as is typical in ecotherms (Arendt 2011), have greater potential fecundity, which may necessitate more remating to satisfy demand for effective fertilization. The influence of longer-term developmental adjustments on remating in females is not well known.

In addition to changing the costs and benefits of female remating in situ, temperature may also potentially influence larger-scale patterns of behavior. One of the most enduring and widespread temperature-derived patterns in nature are latitudinal clines with distinctive equator pole patterns observed for traits such as adult body size, juvenile development time, desiccation resistance, and cold tolerance (Atkinson and Sibly 1997; Gilchrist et al. 2004; Kellermann et al. 2009; Kingsolver 2009). There are also reports of a latitudinal cline in polyandry in 2 North American *Drosophila* species, with northern populations showing higher frequency of polyandry (Pinzone and Dyer 2013; Price et al. 2014). In both species, it is not known whether the clines in polyandry have been shaped and are maintained directly either by environmental temperature or by unique genetic factors. Latitudinal clines in polyandry provide a rare opportunity to examine potential drivers of intraspecific variation in polyandry and to investigate the impact of polyandry on large-scale processes such as gene flow, effective population size, and level of heterozygosity (Taylor et al. 2014).

We examined the influence of temperature on variation in female remating (to represent polyandry more generally) at 2 sites along the latitudinal cline reported in the fly *Drosophila pseudoobscura* (Price et al. 2014). This species ranges across the western North American continent from Guatemala to Canada (Dobzhansky and Epling 1944). Previous research indicates that female remating is consistently higher in northern populations than in those on the US/Mexican border (Price et al. 2014) and varies over at least 13° of latitude (1450 km). Previous work involving 7 populations along the latitudinal cline has also shown that polyandry is genetically determined and highly variable (Price et al. 2008, 2014) and that the percentage of females who remate on the first given opportunity under laboratory conditions strongly correlates with “real” levels of polyandrous behavior in natural populations (Price et al. 2011). However, the influence of temperature on variation in female remating along the observed polyandry cline has not yet been examined. We used a suite of genetically distinct genotypes of females from one northern and one southern population along the cline to examine context (temperature)-dependent variation in polyandry. Our aims here are to 1) determine the influence of temperature on the variation in female remating within populations and the alignment with the larger-scale pattern of polyandry along the cline 2) to examine both the long- and short-term effects of temperature on female remating in *D. pseudoobscura*.

## MATERIALS AND METHODS

### Origin of the flies

**Figure 1:**
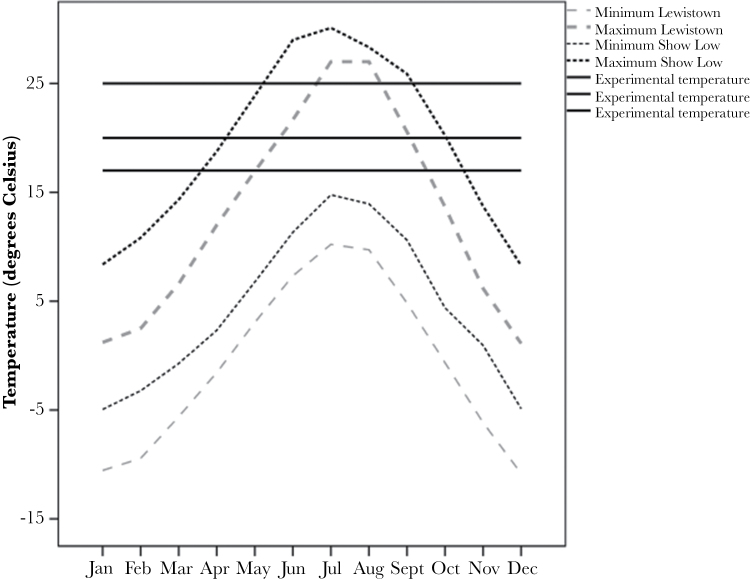
Minimum and maximum monthly temperatures in the 2 geographic locations relative to experimental temperatures. Climate data from 1981 to 2010 is from the archives of the National Oceanic and Atmospheric Administration.

In 2008, we collected wild *D. pseudoobscura* from 2 locations in North America. We collected the northern population at Lewistown, MT (47°03′N, 109°28′W) and the southern population at Show Low, AZ (34°16′N, 110°00′W). We made the collections during the summer season of 2008 and used individual wild-caught females to establish a range of individual genotypes within each population that effectively captured the standing genetic variation in the population. These genotypes are created by inbreeding sibling pairs of a single wild-caught female, which rapidly become homozygous at most alleles, making individuals within genotypes virtually genetically identical while preserving the diversity between genotypes (David et al. 2005). We cultured all of the genotypes under the same routine each generation by selecting a single male and female from newly eclosed offspring and pairing them together for 2 weeks until the appearance of pupating larvae. During this time, sibling pairs were free to mate and oviposit. We discarded adult flies before any new eclosions of offspring to maintain nonoverlapping generations and replicated single sibling pairs 3 times for each genotype to allow for losses. We maintained all flies at 23 °C as this represents a midpoint temperature for the populations, unlikely to unfairly advantage either population (see Figure 1 for annual temperature ranges for both populations) on a 14:10 light:dark cycle and cultured them on 10mL of standard *Drosophila* porridge medium containing water, oats, sugar, brewer’s yeast, and agar, plus a dilute solution of nipagin and propionic acid to inhibit mould and bacteria growth. We conducted the experiment after 45 generations of inbreeding in a common environment to ensure no maternal effects, lag or carry over effects from newly caught individuals, and to minimize adaptation to laboratory conditions (David et al. 2005, but see Griffiths et al. 2005). During this time, we cultured all genotypes under identical conditions to ensure that any effects of inbreeding on plasticity of mating behavior arose from a common stimulus rather than any systematic bias amongst the genotypes. Previous work on inbreeding and plasticity in other *Drosophila* species suggests that effects of inbreeding on the ability to detect clinal variation in plasticity are likely to be minimal (Hoffmann et al. 2001; Griffiths et al. 2005).

### Establishing the baseline levels of polyandry

We assayed females of 26 genotypes (13 from each population) for their propensity to remate to establish genetic variation in polyandry. For this assay, we collected 10 virgin females from each genotype and allowed them to mature for 4 days. At 4 days of age, we paired them with a virgin male from their own genotype and gave them a 2-h period after the incubator lights came on in which to mate. We used this “simulated dawn” period for mating trials because in nature mating activity is absent at the coldest and hottest parts of the day and most common during the crepuscular periods (Dobzhansky and Epling 1944). Because males have very little ability to suppress or coerce female remating behavior in this species (Price et al. 2008, 2010), we considered direct pairing of females with males from their own genotype to be the best logistical handling of the behavioral assay, to also ensure an even representation of male genotypes in the dataset, and to avoid any effects due to females being paired with a nonsibling male after 45 generations of sibling breeding. We maintained the environmental mating temperature at 23 °C to match the standard culture temperature. Following a successful mating, we removed males and left females alone to oviposit at standard culture temperature of 23 °C. After a further 4 days, we moved all females to a fresh vial and presented them with a second virgin male from their own genotype and gave them a further 2-h window in which to remate at 23 °C. We scored the degree of polyandry as the percentage of females that remated from the total number in each genotype tested. This measure of polyandry correlates with female remating rates over longer periods of time in natural conditions (Price et al. 2008, 2011, 2014). We repeated this assay 4 times at 23 °C to measure polyandry in 40 females per genotype. We calculated repeatability across the 26 genotypes, and 4 blocks, using arcsine-square-root–transformed proportions of polyandrous females, to confirm that our method was robust and that there was significant genetic variation in polyandry in our populations (*r* = 0.243, ± standard error [SE] 0.107, *P* = 0.003 (Lessells and Boag 1987; Nakagawa and Schielzeth 2010). To examine whether the latitudinal cline in polyandry is due to plastic responses to temperature or genetic variation in polyandry across populations, we required a range of genotypes representing different levels of polyandry in different populations that were not prebiased toward the cline seen in nature, that is, “highest” in Lewistown and “lowest” in Show Low. To achieve this, we selected the 2 “highest” and 2 “lowest” polyandry genotypes from each population giving us 8 genotypes in total. This ensured that the genetic level of polyandry was variable but independent of the geographic origin of females, giving us the opportunity to examine the latitudinal cline in polyandry using environmental temperature.

### Experimental temperatures and assays

**Figure 2:**
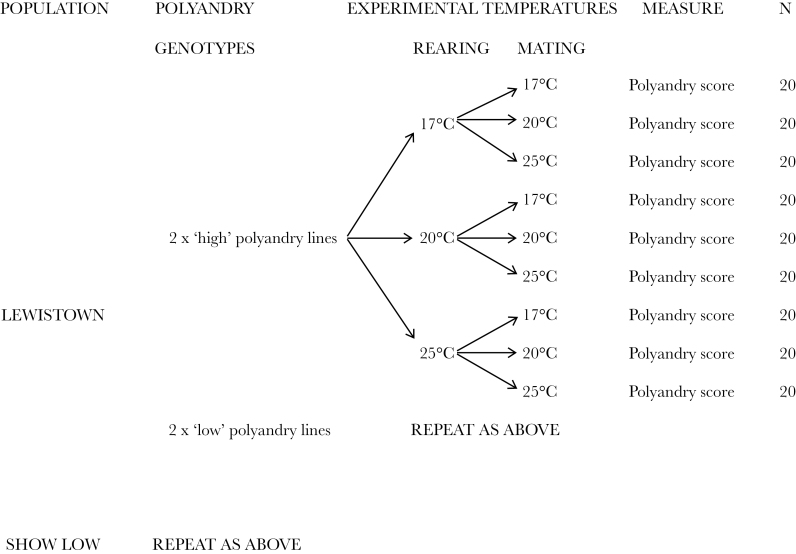
Experimental design to examine the effects of rearing and mating temperatures on female remating in 2 geographical populations of *Drosophila pseudoobscura*.

To calibrate our environmental conditions, we collected long-term climatic data from the archives of the National Climatic Data Centre at the National Oceanic and Atmospheric Administration (www.ncdc.noaa.gov/). We used mean minimum and maximum monthly temperatures compiled over the last 30 years (1981–2010) to construct annual temperature ranges for the 2 geographic locations of *D. pseudoobscura* in our experiment (Figure 1). This confirmed the range of daily temperatures experienced by flies and showed that the 2 populations experience distinct temperatures that form part of a greater temperature cline from north to south. We aimed to replicate a range of temperatures that would explore the variation in female remating over a larger geographic area than that represented by these populations of *D. pseudoobscura* (Figure 1). To examine separately the effects of long- and short-term temperature on female remating, we split each of the 8 genotypes between 3 rearing temperatures (17, 20, and 25 °C) and then used the same temperatures to mate/remate females in a fully factorial design (Figure 2). That is, we reared 60 females from each genotype at one of the 3 rearing temperatures, and then mated 20 females from each genotype/rearing temperature background at each temperature giving a total of 180 females per genotype. It should be noted that *D. pseudoobscura* become infertile at 27 °C (Price et al. 2012) and are largely inactive below 14 °C (Dobzhansky and Epling 1944), so this temperature range extends across the majority of conditions at which they are active in the wild. We maintained all genotypes in their experimental conditions for at least 2 generations before conducting our remating assay to minimize any carryover effects from the standard rearing temperature of 23 °C. For the first mating at a particular temperature (17, 20, or 25 °C), we collected 20 virgin females from each genotype/rearing temperature treatment and allowed them to mature for 4 days. We then presented them with a virgin male, from their own genotype and rearing temperature, and allowed a 2-h window of “simulated dawn” in which to mate as before. We removed males from the vial as soon as possible following a successful mating and returned females alone to their respective rearing temperatures to oviposit. After a further 4 days, we moved all females to a fresh vial and presented them with another virgin male from their own genotype and rearing temperature and allowed a further 2-h window opportunity to remate. For logistical purposes, we conducted all matings on viewing racks within controlled temperature chambers, with all matings in a temperature treatment carried out on the same day during the “simulated dawn” period, that is, we did all matings at 17 °C on the same day and all matings at 20 °C on a separate day etc. We again scored degree of polyandry as the percentage of females that remated in the 2-h window from the total number of females in that genotype/temperature treatment that were given the opportunity to remate. We also examined more closely some of the behavioral components of mating in each of the experimental treatments. For each female, we recorded the mating latency (time from the male being placed in the vial to the start of copulation) and copulation duration of the first mating. This was to explore the possibility that male activity or certain male phenotypes were driving female decisions to remate. All statistics and graphics were created using SPSS v 20 (IBM Statistics).

## RESULTS

We conducted a preliminary analysis of the average baseline level of female remating in the 26 genotypes from the 2 populations to confirm that polyandry was genetically variable between the 2 populations in a common garden rearing and mating environment of 23 °C. We conducted an analysis of variance of polyandry, using arcsine-square-root–transformed proportions of remated females, with population as a random factor. Mean degree of polyandry in the Lewistown population (mean polyandry: 20%, ±SE 2, *N* = 51) was significantly higher than that in Show Low (mean polyandry: 15%, ±SE 2, *N* = 52) (univariate analysis of variance [Anova] of polyandry by population: *F*_1, 101_ = 3.818, *P* = 0.05), which is consistent with the latitudinal differences in polyandry reported in Price et al. (2014). We then reanalyzed the 8 genotypes chosen for the experimental study and confirmed that they also varied significantly in level of polyandry (univariate Anova of polyandry by 8 genotypes: *F*_7, 24_ = 5.898, *P* = 0.000), which was unsurprising as they had been chosen to represent the extreme levels of polyandry. Finally, we examined whether the 8 genotypes chosen were prebiased with respect to the latitudinal cline (i.e., highest average polyandry in Lewistown, lowest average polyandry in Show Low) and confirmed that genotypes belonging to either Lewistown (mean polyandry: 23%, ±SE 5, *N* = 16) or Show Low (mean polyandry: 17%, ±SE 3, *N* = 16) did not differ significantly from each other (univariate Anova of polyandry in 8 genotypes by population: *F*_1, 30_ = 0.60, *P* = 0.455), again unsurprising as they had been chosen to not differ by population. This essentially confirmed that we were using 2 populations that represented different areas of the latitudinal cline in polyandry, and had selected a sample of genotypes from each population that showed genetic variation in polyandry but were neutral with respect to latitude with which to test the effects of temperature on the cline in polyandry.

**Figure 3:**
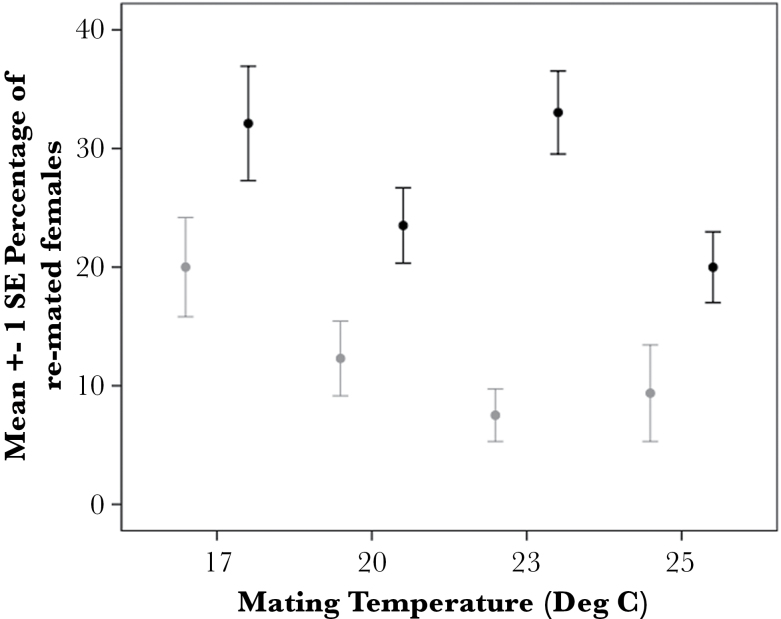
The mean (± 1 SE) percentage of remated females (polyandry) in the highest polyandry lines (black lines) and lowest polyandry lines (gray lines) scored across 4 mating temperatures. Data from all genotypes are pooled within groups and data from the original baseline assay at 23 °C is included for comparison in the figure only.

**Figure 4:**
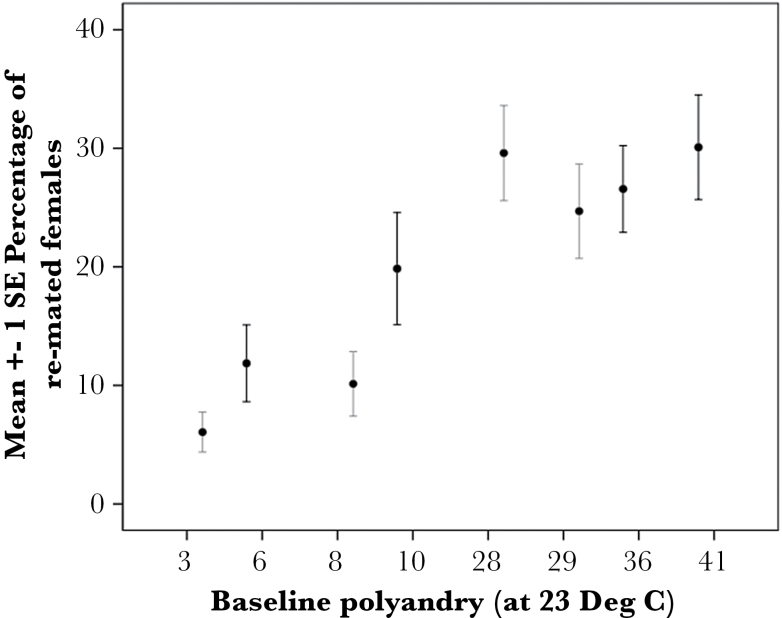
The mean (± 1 SE) percentage of remated females (polyandry) in Lewistown (black lines) and Show Low (gray lines) females across all temperature treatments. Individual genotypes are ranked and labeled by their baseline degree of polyandry that was scored in the first assay of all 26 genotypes at 23 °C.

Our first objective was to establish the influence of temperature on the variation in female remating across genotypes and the alignment with the latitudinal cline in polyandry reported in *D. pseudoobscura* (Price et al. 2014). We used binary logistic regression with individual females scored as 1 (remated) or 0 (not remated) to examine the strength of relationship between each variable (genotype, mating temperature, rearing temperature) and remating (Jaegar 2008). We found that temperature experienced at the time of mating significantly influenced female remating in a pattern consistent with the latitudinal cline reported (Table 1, Figure 3). Overall, pooling across the 8 genotypes and 3 rearing temperatures, more females remated at the lowest mating temperature (mean polyandry 17 °C: 26%, ±SE 3, *N* = 24) than at the highest mating temperature (mean polyandry 25 °C: 15%, ±SE 2, *N* = 23) (Figure 3). However, we also found that genotype retained a significant influence over female remating, with genotypes that scored as “high” polyandry remaining high, and vice versa, irrespective of the temperature treatment (Table 1, Figure 4; repeatability: *r* = 0.55, ±SE = 0.181, *P* = 0.000). We found no significant interactions between genotype and mating temperature (univariate Anova: *F*_14, 44_ = 1.193, *P* = 0.314). This concurs with our previous work demonstrating significant heritability in polyandry (Price et al. 2014) and indicates that all females from all genotypes responded to the influence of temperature in the same way, that is, remated more at cooler temperatures (Figure 3).

**Table 1:** Logistic regression of polyandry (females remated = 1, not remated = 0) across 8 genotypes, 3 rearing temperatures, and 3 mating temperatures, with genotype and mating and rearing temperatures as predictors

Model includes	*B*	±SE	*P*
Constant	0.312	0.696	0.654
Genotype	2.628	0.549	0.000
Mating temperature	−0.083	0.024	0.000
Rearing temperature	−0.029	0.024	0.226
Model chi square (df 3) = 36.72, *P* = 0.000			
Model *R* ^2^ = 0.032; *N* = 1121			

*B* gives the slope of the regression of each individual variable (along with its SE and significance level), whereas chi square gives the significance of the overall model. df, degrees of freedom.

Our second objective was to investigate whether variation in female remating was a result of long- (developmental) or short-term responses to temperature. We found no evidence that rearing temperature had any effect on remating (Table 1), so females did not alter remating after developmental adjustments and only responded behaviorally to the temperature at the point of mating. We measured activity before and during copulation and found that both were significantly, negatively related to mating temperature (mating latency: *r* = −0.098, *P* = 0.001, *N* = 1118; copulation duration: *r* = −0.457, *P* = 0.000, *N* = 1101), so pairs of flies in cooler environments took longer to initiate copulation and remained in copula for a longer duration. However, neither of these variables, measured during the first mating, predicted whether females would remate (Table 2).

**Table 2:** Logistic regression of polyandry (females remated = 1, not remated = 0) across 8 genotypes, 3 rearing temperatures, and 3 mating temperatures, with mating latency and copulation duration of the first mating as predictors

Model includes	*B*	±SE	*P*
Constant	−1.54	0.138	0.000
Mating latency	0.003	0.004	0.499
Copulation duration	0.019	0.016	0.236
Model chi square (df 2) = 1.893, *P* = 0.388			
Model *R* ^2^ = 0.002; *N* = 1100			

*B* gives the slope of the regression of each individual variable (along with its SE and significance level), whereas chi square gives the significance of the overall model. df, degrees of freedom.

Taken together, these results show that there is no influence of temperature experienced as a larva on remating in adult females and only a moderate effect of temperature experienced during the mating period itself. Temperature-based variation in female remating appears to coincide with the genetic pattern of polyandry reported previously and is consistent with the overall latitudinal pattern that remating is more common in cooler, northern populations.

## DISCUSSION

### The influence of temperature on female remating

We used females from a naturally occurring latitudinal cline in polyandry in *D. pseudoobscura* to examine whether temperature can shape patterns of female remating. We found that females of all genotypes were more likely to remate at cooler temperatures, irrespective of their genetic background. Mean frequencies of polyandry ranged from 26% at the coldest temperature (17 °C) to 15% at the hottest temperature (25 °C). This effect is in direct contrast to the patterns emerging from other insect studies that report *more* female remating at *higher* temperature (e.g., Saeki et al. 2005; Kindle et al. 2006; Katsuki and Miyatake 2009; Best et al. 2012). Our measures of mating latency and copulation duration showed that females copulated after a longer courtship and for a longer amount of time at cooler temperatures, which matches other work (e.g., Martin and Hosken 2002; Katsuki and Miyatake 2009), yet neither of these measures directly influenced female remating.

Mechanisms to explain patterns of higher female remating at cooler temperatures are less intuitive than those to explain why high temperature might increase polyandry. The most likely explanation is either that polyandrous behavior is itself less costly under cooler temperatures or that polyandry serves to reduce costs that are increased at cooler temperatures. One possibility is that desiccation risk is lower at cooler temperatures so that individuals can remain active much longer toward the hottest part of the day and increase the chances of finding multiple mates. We did not directly measure humidity in our experiments, but Dobzhansky and Epling (1944) report data from trapping rates in natural populations where humidity was measured. They showed that adults were trapped at a range of humidity from 11% to 100% and concluded that humidity itself was not a strong environmental determinant of adult activity. Alternatively, females could remate to reduce the costs of harassment from males that are more active at lower temperatures although we note that mating latency (time taken for females to accept males for mating) in our experiment was longer at lower temperatures. Other biological processes that directly regulate female postmating refractory periods, such as the presence of sex peptides in the reproductive tract, are more likely to delay female remating at cooler temperatures, as cooler temperatures slow the breakdown of male-derived seminal peptides (Best et al. 2012). Further explanations that revolve around the potential for cooler temperatures to reduce the ecological costs of remating, for example, risk of sexually transmitted infections or predation risk, were not directly tested here, and so are less likely to explain our results than intrinsic biological processes in these circumstances. At present, we know of no evidence that supports a specific mechanism linking colder temperature and female remating frequency in this species.

We also explored the possibility that female remating would be influenced by longer-term responses to temperature. Under the temperature–size rule (Atkinson and Sibly 1997; Arendt 2011; Forster et al. 2011; Forster and Hirst 2012), females developing at cooler temperatures were predicted to mature at larger body sizes than their genetic counterparts in warmer conditions, with the assumption that larger females would remate more to capitalize on their higher potential fecundity. In other work, we have shown body size to be significantly influenced by temperature, with larger females emerging from cooler temperatures (Taylor et al. 2015), so females from northern populations are expected to be larger. However, we also found that females from the southern population are *genetically* larger than those from the north, potentially obscuring a clear body size–polyandry relationship. We suggest that female remating in this case were not strongly influenced by the potential fitness benefits associated with larger body sizes resulting from cooler developmental temperatures. Other developmental processes that could potentially influence female remating include temperature-induced variation in male fertility in the different genotypes. For example, *D. pseudoobscura* are known to be infertile when maintained at temperatures of 27 °C or more (Anderson 1973; Price et al. 2012). We used this benchmark to set an upper limit to our experimental temperatures (26 °C). However, if males from northern populations had a lower temperature threshold of fertility this could provide an explanation for higher remating in females paired with such males. In this case, we do not think this is a strong candidate mechanism to explain our results, as developmental temperatures were not significantly associated with female remating rates and if northern males experienced infertility at a lower temperature threshold, then we would expect to see higher proportions of remating in northern females kept at higher temperatures, which is opposite to the pattern we found.

The general response to temperature by all females fits well with the current latitudinal pattern of higher frequency of polyandry observed in northern, cooler, populations (Price et al. 2014). However, despite this overall response to temperature, females retained their relative ranks of high and low polyandry within each population. In other words, temperature produced relatively similar responses in individual females, so that variation in female remating observed in the original baseline assay at standard temperature (23 °C) was preserved. It is likely, therefore, that patterns of variation in female remating are more strongly determined by underlying genetic factors than by plastic responses to the environment in this species. This may also help to explain the contrast of our findings with other studies of temperature and polyandry.

### A genetic explanation for the latitudinal cline in polyandry

By far our most interesting result was that polyandry remained strongly determined by the genotype of females, despite females being reared at very different temperatures and significantly adjusting their mating behavior to temperature. Mean female remating ranged from 22% in the 8 genotypes from northern populations to 18% in the 8 genotypes from the southern population, and although these averages were not significantly different (we had deliberately selected them to avoid this bias), this pattern remained despite the environmental influence. Moreover, previous work has shown that the genetic differences in female remating are almost entirely due to the females themselves, with very little variation in female mating frequency due to males either suppressing or coercing female remating (Price et al. 2008, 2010). Estimates of migration and gene flow in this species show that populations have not diverged simply from random drift or historic factors as flies can move considerable distances and populations interbreed (Schaeffer and Miller 1992). The most likely explanations for a genetic basis to polyandry are localized selection, and/or unique genetic factors such as chromosome inversions (Herrera et al. 2014).

There is a suite of classic models of selection that could account for the genetic variation in polyandry within the populations we observed (Arnqvist and Nilsson 2000; Jennions and Petrie 2000; Simmons 2001, 2003, 2005). Previous work in this species suggests that females may gain direct fecundity benefits from remating, possibly due to sperm replenishment, and that sexual conflict over female mating frequency may be especially costly for monandrous females (Turner and Anderson 1983; Crudgington et al. 2005). We collected fecundity data on a subsample of females from the 20 °C mating treatment in preparation for further work into the costs and benefits of polyandry (Supplementary Material). We found no significant fecundity differences in females from the 2 populations mating either monandrously or polyandrously that would indicate direct selection on variation in female remating behavior, but this possibility needs to be fully explored. Whether other forms of selection can maintain average rates of female remating across populations to produce the latitudinal cline observed is not yet known and is the subject of further work.

Alternatively, variation in polyandry could be maintained across latitude by the system of chromosome inversions known to occur in *D. pseudoobscura* (Meisel and Schaeffer 2007), which could effectively “trap” regions of the genome that control female remating within inverted regions. We have investigated the relationship between inversion karyotype and female remating frequencies in other genotypes from the same populations as those used in this study, but found no direct link between the two (Herrera et al. 2014). However, chromosome inversions could influence genetic variation and behavioral plasticity more generally, which could influence female remating indirectly through the effect on related traits (Dingemanse and Wolf 2013). In other words, karyotype may not directly control female remating itself, but control a trait affecting female remating such as desiccation resistance.

## SUMMARY

We asked whether a large-scale environmental variable such as temperature could explain variation in female remating and, by extension, a latitudinal cline in polyandry in *D. pseudoobscura*. We found that females remated more at cooler temperatures but that temperature was a secondary influence after the dominant role of genotype in variation in female remating across the cline.

## SUPPLEMENTARY MATERIAL

Supplementary material can be found at http://www.beheco.oxfordjournals.org/

## FUNDING

This work was supported by the Natural Environment Research Council (grant number NE/I0277/11/1 to N.W. and T.A.R.P.).

## Supplementary Material

Supplementary Data
