# Supplementary material for: Temperature can shape a cline in polyandry, but only genetic variation can sustain it over time
Source: Behav Ecol. 2015 Oct 25;27(2):462–9. doi: 10.1093/beheco/arv172 (PMC4797379; doi:10.1093/beheco/arv172)
Supplement: Supplementary Data [file supp_27_2_462__index.html]

Temperature can shape a cline in polyandry, but only genetic variation can sustain it over time — Temperature can shape a cline in polyandry, but only genetic variation can sustain it over time — Temperature can shape a cline in polyandry, but only genetic variation can sustain it over time — Supplementary Data 

# Temperature can shape a cline in polyandry, but only genetic variation can sustain it over time

## Supplementary Data

Data files

- Supplementary Data - Supplementary Data
