# Supplementary material for: Temperature can shape a cline in polyandry, but only genetic variation can sustain it over time
Source: Behav Ecol. 2015 Oct 25;27(2):462–9. doi: 10.1093/beheco/arv172 (PMC4797379; doi:10.1093/beheco/arv172)
Supplement: Supplementary Data [file supp_arv172_Behav_Ecol_Supp_Mat.doc]

**Behavioral Ecology – Supplementary Material**

**Background**

To further explore the possible basis for our main result that polyandry is governed by genetic variation, rather than large scale environmental variables, such as temperature, we collected fecundity data in a subsample of females to use as pilot data for a full scale experiment into the costs and benefits of polyandry. We used females from the final mating assay conducted at 20^o^C to standardize fecundity according to mating temperature, known to significantly affect behaviour. We used females across all rearing temperatures to achieve the sample sizes for the assay, and as our results showed, this did not affect female mating behaviour.

**Methods**

From our mating assay conducted at 20^o^C we took a subsample of females from each genotype; five that had behaved monandrously and five that had behaved polyandrously. We transferred them to fresh vials containing 10ml of standard *Drosophila* medium for 4 days (after first mating), then another 4 days (after second mating) and then every 3-4 days, up to a total of 18 days from first mating. All females were removed at this point. We kept all vials at standard housing conditions of 14:10 hrs light: dark cycle and 23^o^C throughout. We then counted all of the offspring that eclosed from all of the vials at a fixed time point, which was seven days after the first fly in each vial eclosed.

**Results**

We found that total fecundity was marginally non-significantly (P=0.056) influenced by the behaviour of females in each genotype – with monandrous females having marginally higher fecundity than polyandrous females (Table 1 and Figure S1). Perhaps of most interest, is that the degree of polyandry scored in the baseline assay at 23^o^C, although not significant, showed a negative trend with the total fecundity, regardless of female re-mating behaviour at 20^o^C (Figure S1). In other words, genotypes that were originally scored as being ‘high’ polyandry had generally lower fecundity than genotypes that were scored as ‘low’ polyandry.

We found no difference in fecundity in females from different populations that behaved monandrously or polyandrously (Table 2 and Figure S2). However, this is not surprising, as the genotypes were selected to represent similar population level averages in behaviour. We note that this fitness data was taken for only a small-subset of females representing only eight genotypes per population and that larger sample sizes will be needed to fully investigate the link between polyandry, fitness and genetic background.

Table 1. ANOVA of total fecundity in females from all genotypes that behaved either monandrously or polyandrously, with the baseline level of polyandry scored at 23^o^C included as a covariate.

| *Total Offspring* | Df | F | P |
| --- | --- | --- | --- |
| Behaviour (M or P) | 1, 68 | 3.771 | .056 |
| Polyandry in baseline assay at 23^o^C | 1, 68 | 3.387 | .070 |

Table 2: Two-way ANOVA of total offspring eclosed in females from Lewistown or Show Low that behaved either monandrously or polyandrously.

| Variable | df | F | p |
| --- | --- | --- | --- |
| *Behaviour – monandrous vs polyandrous* | 1 | 5.889 | .249 |
| *Population – Lewistown vs Show Low* | 1 | 9.782 | .197 |
| *Behavior x population* | 1 | 0.268 | .607 |
| *Error* | 51 |  |  |


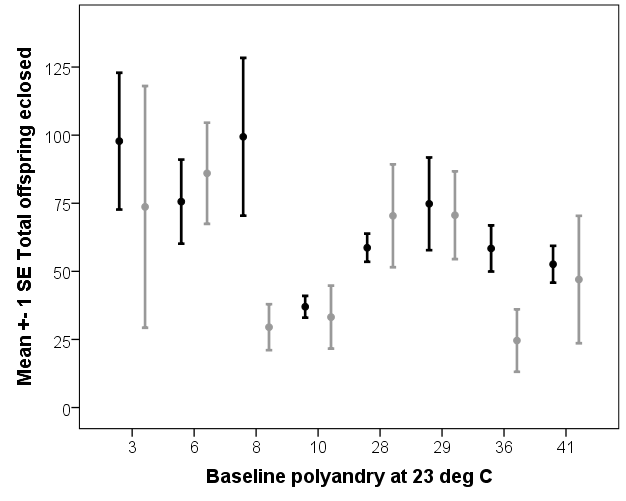


Figure S1. Total fecundity of females from each genotype that behaved either monandrously (black bars) or polyandrously (grey bars).


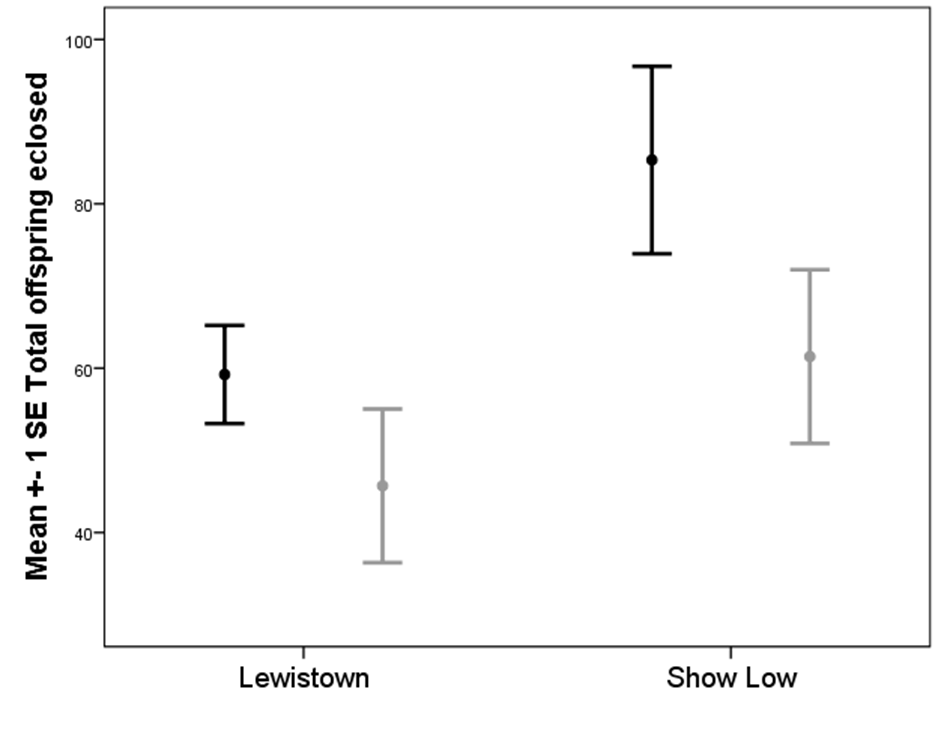


Figure S2. Fecundity in females from Lewistown and Show Low (pooled across four genotypes in each population) that behaved either monandrously (black lines) or polyandorusly (grey lines) (mated at 20^o^C).
